# Spring-loaded inverted pendulum goes through two contraction-extension cycles during the single-support phase of walking

**DOI:** 10.1242/bio.043695

**Published:** 2019-05-16

**Authors:** Gabriel Antoniak, Tirthabir Biswas, Nelson Cortes, Siddhartha Sikdar, Chanwoo Chun, Vikas Bhandawat

**Affiliations:** 1 Department of Biology, Duke University, Durham, NC 27708, USA; 2 Department of Physics, Loyola University, New Orleans, LA 70118, USA; 3 Department of Bioengineering, George Mason University, Fairfax, VA 22030, USA

**Keywords:** Biomechanics, Locomotion, Spring-loaded inverted pendulum

## Abstract

Despite the overall complexity of legged locomotion, the motion of the center of mass (COM) itself is relatively simple, and can be qualitatively described by simple mechanical models. In particular, walking can be qualitatively modeled by a simple model in which each leg is described by a spring-loaded inverted pendulum (SLIP). However, SLIP has many limitations and is unlikely to serve as a quantitative model. As a first step to obtaining a quantitative model for walking, we explored the ability of SLIP to model the single-support phase of walking, and found that SLIP has two limitations. First, it predicts larger horizontal ground reaction forces (GRFs) than empirically observed. A new model – angular and radial spring-loaded inverted pendulum (ARSLIP) – can overcome this deficit. Second, although the leg spring (surprisingly) goes through contraction-extension-contraction-extensions (CECEs) during the single-support phase of walking and can produce the characteristic M-shaped vertical GRFs, modeling the single-support phase requires active elements. Despite these limitations, SLIP as a model provides important insights. It shows that the CECE cycling lengthens the stance duration allowing the COM to travel passively for longer, and decreases the velocity redirection between the beginning and end of a step.

## Abstract (precis)

**Summary:** We quantitatively evaluate a simple model for human walking based on two-spring and find that this model describes many features of human walking.

## INTRODUCTION

Legged locomotion is complex; therefore, many approaches to legged locomotion focus on the motion of the animal's center of mass (COM) rather than on the detailed dynamics of each joint (Full and Koditschek, 1999). Traditionally, walking and running were described using different mechanical systems: the stiff-legged inverted pendulum (IP) was used as a model for walking (Griffin et al., 2004; Usherwood, 2005; Buczek et al., 2006), while the spring-loaded inverted pendulum (SLIP), with its compliant legs, was used as a model for running (Blickhan, 1989; McMahon and Cheng, 1990; Blickhan and Full, 1993; Ahn et al., 2004; Daley et al., 2007; Nishikawa et al., 2007; Schmitt, 1999). The recent realization that legs are compliant during walking has (Lee and Farley, 1998; Buczek et al., 2006) led to the development of the double SLIP (DSLIP) model, in which each leg of a biped is modeled as a spring. DSLIP extends SLIP with a double-support phase during which the COM is supported by two ‘springy’ legs (Geyer et al., 2006; Rummel et al., 2010). These studies suggest that simple mechanical models can serve as conceptual models for locomotion. However, a central question is whether these models describe locomotion well enough to serve not just as conceptual models, but as quantitative models for locomotion that can be used to understand the control of locomotion by the nervous system (Holmes et al., 2006).

Current versions of DSLIP models have many deficiencies which make it unlikely that they can serve as a quantitative model for walking in their current form. As noted by the authors themselves, DSLIP finds stable gaits only for a limited range of speeds (Geyer et al., 2006; Lipfert et al., 2012), and predicts within-step variations in COM height and ground reaction forces (GRFs) (Lipfert et al., 2012; Hubel and Usherwood, 2015) that are larger than those observed experimentally. DSLIP is also unable to reproduce experimentally observed stance durations; predicted stance durations are always shorter than those observed empirically. These deficiencies of DSLIP might arise from two sources. First, we have already shown that replacing each leg with a single linear spring is an oversimplification; one also needs to account for tangential forces (Biswas et al., 2018). Second, it is possible that the right parameter regime that models experimentally observed dynamics most accurately, particularly in the case of walking, have not been discovered. Thus, it is likely that either a more rigorous approach to parameter search, or a different dynamical model, or a combination of the two would yield a quantitative model for walking.

**Fig. 1.**
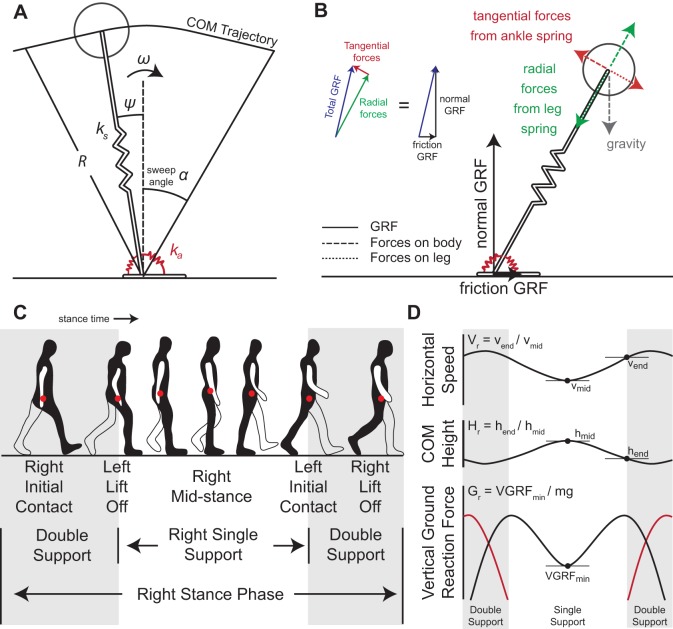
**ARSLIP model and gaitspace constraint description.** (A) The ARSLIP template contains a leg spring of stiffness *k_s_* and an ankle angular spring of stiffness *k_a_* that resists motion away from the vertical orientation of the leg. (B) The interaction between the ground, leg and the body in the ARSLIP model. The GRFs that the leg receives are denoted by the black arrows. The forces are then transmitted to the body by the leg in the form of the dashed tangential and radial forces, colored red and green, respectively. The equal and opposite forces that the leg receives from the body (red and green dotted lines) cancel the GRFs as shown in the inset, since the leg is massless. (C) Schematic for the stance period of gait cycle for the right leg. The red dot represents the COM. During single-support, a single leg is in contact with the ground, which occurs in the middle of the stance phase. (D) Constraints on the gaitspace. The velocity constraint is the ratio of the horizontal velocity at the end of single-support to the mid-stance horizontal velocity. The height constraint is the ratio of the COM height at the end of single-support to the mid-stance height of the COM. The vertical ground reaction force constraint is the minimum ground reaction force during single-support normalized by body weight.

The dynamical model proposed here is the angular and radial spring-loaded pendulum (ARSLIP) model (Fig. 1A), and is aimed at improving the ability of SLIP to model ground reaction forces (GRFs) experienced by animals during walking. Animals receive GRFs in the form of normal and frictional forces that act vertically and horizontally, respectively (Fig. 1B). SLIP assumes that these two forces adjust such that the total GRF is always aligned along the effective leg (radial forces). There is no *a priori* reason that GRFs should always be along the leg, and indeed several studies have indicated otherwise (Maus et al., 2010; Müller et al., 2017). Like SLIP, ARSLIP also has a point mass supported by a massless leg. ARSLIP extends SLIP by providing a mechanism for modeling tangential forces. The tangential forces modeled by ARSLIP are restorative and change sign at mid-stance, see Fig. 1; these restorative forces were shown to be important for walking (Biswas et al., 2018). There are other models for tangential forces. But most of these models only produce unidirectional torques (Seipel and Holmes, 2007; Ankarali and Saranli, 2010; Shen et al., 2014). Some models whose intended purpose is different to ours do produce tangential forces similar to the ones produced in ARSLIP. One model adds a roller foot to a springy leg (Whittington and Thelen, 2009; Kim and Park, 2011). Another model proposes that the COM is below the point through which the force acts, resulting in restoring forces (Maus et al., 2010; Sharbafi and Seyfarth, 2015). Both of these methods help in overcoming some limitations of SLIP, but because the radial and tangential forces are not independent, many weaknesses remain. In ARSLIP, the radial and tangential forces are independently tuned by two independent springs. Conceptually, a simple method to visualize the model is to think of an angular spring connecting the foot to the leg as depicted in Fig. 1A. More details about the model can also be found in the Supplementary Information (Model formulation).

Our approach to evaluating a model is different from previous studies in two respects: the first point is conceptual. In contrast to most traditional approaches to simplified ‘template’ models which are passive, we conceptualize our models as passive within a step. That is, the model parameters are the parameters that are controlled by an animal during a step.

The second point relates to defining what constitutes a successful model. Most studies focus on a single aspect of locomotion such as GRFs; our approach focuses on three important aspects of locomotion: GRFs, COM kinematics and stance duration. That is, a successful model must produce realistic GRFs within the constraints of experimentally observed COM kinematics over experimentally observed stance duration. These three constraints are rarely satisfied (Maus et al., 2010, 2014; Lipfert et al., 2012) simultaneously in most studies of locomotion. Without satisfying the constraints on force, kinematics and duration simultaneously, the problem is under-constrained: the COM height determines the natural timescale of the system 
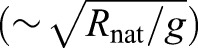
 and, in understanding locomotion, one important consideration is how does the stance duration compare to the natural time constant? Allowing *R*_nat_ to be arbitrary ignores this important constraint. It is precisely because of the presence of the gravitational time-scale that slow walking is a challenge that therefore has to be addressed in any realistic model of locomotion (Biswas et al., 2018).

This manuscript focuses on the single-support phase of walking (Fig. 1C). As mentioned earlier, walking can be divided into two phases: a relatively short double-support phase, during which both legs are on the ground, and the longer single-support phase. Neither the stiffness nor the natural length of the leg remains constant during the entire stance duration. During the double-support phase, the ankle is flexible; as the leg proceeds to its single-support phase, the ankle becomes stiffer. Moreover, a very influential series of studies have described the double-support phase as a transition phase (Donelan et al., 2002; Adamczyk and Kuo, 2009) that is unlikely to be described using pendular models. Therefore, as a first step to obtaining a biomechanical model of the entire walking cycle, here we study the mechanics of the single-support phase. Using non-dimensional analysis, we show that SLIP can model the single-support phase of walking, and ARSLIP expands the parameter range over which realistic walking can be modeled. Surprisingly, fitting SLIP to empirical walking data shows that the stance leg goes through a contraction-expansion-contraction-expansion (CECE) cycle during single-support. This cycling appears important for reducing the extent to which velocity vectors have to be redirected during the double-support phase, and increases the stance duration and makes it possible to travel passively for longer distances.

## METHODS AND RESULTS

### Non-dimensional analysis of SLIP and ARSLIP shows that ARSLIP expands the parameter space over which biologically observed gaits are possible

Successful models of locomotion have to be constrained not only by the average COM kinematics, but also by the within-step fluctuations in the COM kinematics because these constraints are typically observed during locomotion. For example, the small changes in COM height that are observed during a step (Fig. 1D) place stringent constraints on spring stiffness. In this section, we will define constraints on the COM height, speed and GRFs (Fig. 1D) that must be satisfied by a successful model. We refer to the parameter subspace within which these constraints are satisfied as the ‘gaitspace’. This analysis extends a previous analysis of SLIP (Biswas et al., 2018) by including a new model, ARSLIP, and by including GRF in our analysis.

#### Constraints on COM height and speed, as well as GRF

The COM height for a human during walking changes by less than 10% of its leg length (Lee and Farley, 1998). The constraints on height (Biswas et al., 2018) was implemented using the ‘height ratio’, *H_r_*≡(Beginning COM Height)/(Mid-stance COM height), see Fig. 1D. The constraint on *H_r_* was imposed as follows:(1)
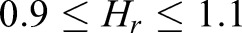


Changes in the horizontal speed of the COM lie within 25% of the mean (Cavagna et al., 1977; Blickhan and Full, 1987; Farley and Ko, 1997). and was implemented by introducing a ‘velocity ratio’, *V_r_*≡(Beginning horizontal velocity)/(Mid-stance horizontal velocity). The constraint on speed was imposed as follows:(2)
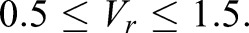
A relatively large band of speed is allowed because the speed fluctuations are large for some animals. Only symmetric walking gaits are considered so that the height and horizontal speed at the beginning and end of a given stance phase is the same.

We imposed two constraints on VGRF. First, VGRF rarely goes below 30% of the weight of the animal (Hubel and Usherwood, 2015; Bishop et al., 2018). Constraint on VGRF was implemented through the ‘VGRF ratio’, *G_r_*≡(minimum VGRF)/*mg*, and we imposed the constraint:(3)
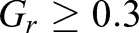
For mammals, including humans (Cavagna and Margaria, 1966; Lee and Farley, 1998), certain birds (Bishop et al., 2018; Daley and Birn-Jeffery, 2018) and some quadrupeds (Shine et al., 2017; Bobbert et al., 2007; Kaya et al., 2006) the VGRF has a mid-stance minimum and is flanked on each side by two local maxima thereby producing a characteristic M-shape (see Fig. 1C). This constraint was implemented by ensuring that the VGRF, *F_y_*, has a minimum (‘VGRF convexity’) at the mid-stance (although we note that the VGRF are not always symmetrical), where *t* represents time:(4)
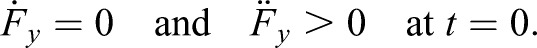
Realistic walking gaits must satisfy (Eqns 1–4).

#### Models, evolution equations and dimensionless parameterizations

The SLIP and ARSLIP models are shown in Fig. 1. SLIP is a special case of ARSLIP with the angular-spring stiffness set to zero. The governing differential equations for single-support of ARSLIP can be obtained from energy considerations (see Supplementary Information, Model formation), and are given by:(5)
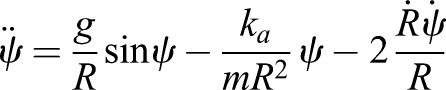
(6)
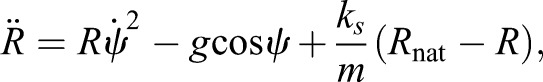
where *ѱ*(*t*) is the angle the leg makes with the vertical mid-stance position, *R*(*t*) is the effective leg length from the COM to the contact point during mid-stance, *k_a_* is the stiffness of the angular spring, *k_s_* is the stiffness of the leg spring, *g* is the gravitational acceleration constant, and *R*_nat_ is the uncompressed length of the effective leg. The evolution of the COM from the mid-stance position is assumed to be symmetric, so the initial conditions for the differential equation are: *R*(0) = *R*˳, *Ṙ*(0) = 0, *ѱ*(0) = 0, and 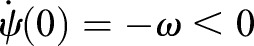; the mid-stance is chosen to occur at *t* = 0. Thus a symmetric gait is specified by six parameters: *k_s_*, *k_a_*, *R*_nat_, *R*˳, *ω* and *α*, where *α* denotes the ‘sweep angle’ (Fig. 1A). To perform analysis that can be generalized, we recast the evolution equation in terms of dimensionless variables:(7)
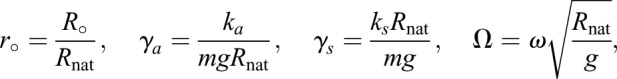
where *r*˳ is the non-dimensional vertical height of the COM at mid-stance, γ*_a_*, γ*_s_* represent the non-dimensional angular and radial spring constants, respectively, and Ω is the non-dimensional angular speed at mid-stance. The evolution of the COM in non-dimensional form is given by Eqns 8 and 9 below:(8)
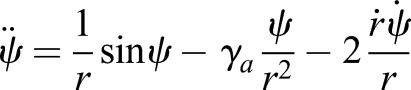
(9)
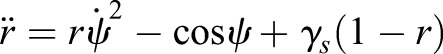


Where *r* = *R/R*_nat_ is the non-dimensional radial coordinate and the differentiations are now with respect to the non-dimensional time 
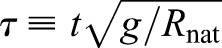
. As is well known, working with non-dimensional quantities also reduces the number of independent parameters making it easier to explore the full parameter space. In terms of dimensionless variables, SLIP has four parameters, γ*_s_*, Ω, *r*˳ and *α*. For ARSLIP, one needs to add another in γ*_a_*.

#### Gaitspace for SLIP and ARSLIP

To determine the gaitspace, MatLab simulations were performed using the non-dimensional Eqn 8 and 9. Every parameter set that satisfied the constraints – set out in height ratio (Eqn 1), velocity ratio (Eqn 2), VGRF ratio (Eqn 3) and profile (Eqn 4) – gave rise to, according to our definition, a realistic gait. For these gaits, we also obtained the Froude number (Fr),(10)
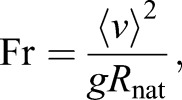
which characterizes the walking speed, ⟨*v*⟩ being the average walking speed over the step.

**Fig. 2.**
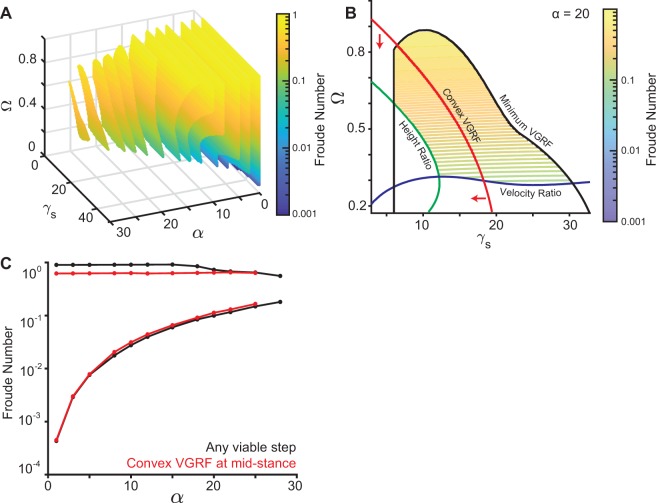
**Non-dimensional analysis of SLIP model.** (A) A sequence of 2D slices each corresponding to one value of *α*. The colored area is allowed under the constraints described in Fig. 1D. Colors represent the Froude number. The vertical mid-stance height *r*˳ is fixed at 0.95. (B) 2D contour plot of the *α* = 20 degree slice for *r*˳ = 0.95 as in A. Black represents the VGRF criterion boundary, blue the velocity criterion boundary, and green the height criterion boundary. The red line separates the concave and the convex VGRF regions, with the convex region (mid-stance minimum) denoted by the red arrows. (C) Range of Froude number supported under the constraints in Fig. 1D. The red line represents additionally meeting the convex VGRF criterion. For each *α*, γ*_s_* and Ω were varied to obtain the slowest and fastest steps. *r*˳ was fixed to 0.95 like in A.

Fig. 2A shows the region where constraints (Eqns 1–4) are satisfied for SLIP. Fig. 2A was constructed by combining a series of two dimensional slices each corresponding to different values of *α*. Fig. 2A uses *r*˳ = 0.95, a value close to that observed in humans (see section ‘Three mechanisms for speed control: changes in step amplitude, radial spring constant and angular spring constant’). The results are similar for different *r*˳ (Figs S1 and S2). Fig. 2B shows one of the two-dimensional slices (for *α* = 20°, an angle that is typical for humans), and shows how each of the constraints in Eqns (1–4) affect the gaitspace.

There are two notable features of the SLIP gaitspace. First, as *α* increases, the SLIP gaitspace shrinks, decreasing the range of Fr allowed. Slow speeds (below Fr = 0.1) are not allowed unless the step length is short. The reason is as follows: in SLIP, there has to be an approximate balance between the radially outward centrifugal and tension forces on one hand, and the radially inward component of gravity on the other. For slow speeds, since Ω that controls centrifugal force is small, the γ*_s_* that is responsible for the spring force must be large. However, heuristically one expects the oscillation time, 
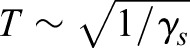
, and thus if γ*_s_* is too large, the leg relaxes to its natural length too quickly for the leg to sweep through the given stance angle. This finding is indeed consistent with previous findings in Biswas et al. (2018) and Kim and Park (2011). Fig. 2C shows the range of allowed speeds as a function of *α*.

Another important feature is that, unexpectedly, a single springy leg can produce the M-shaped GRF. In previous work on DSLIP and its variant the peaks of the M-shape invariably occur during the double-support phase (Geyer et al., 2006; Rummel et al., 2010), instead of during the single-support phase. This ability of SLIP is critical to its appropriateness as a model for walking because empirical GRF peaks are invariably during the single-support phase (Cavagna and Margaria, 1966; Inman, 1966; Buczek et al., 2006; Kim and Park, 2011).

**Fig. 3.**
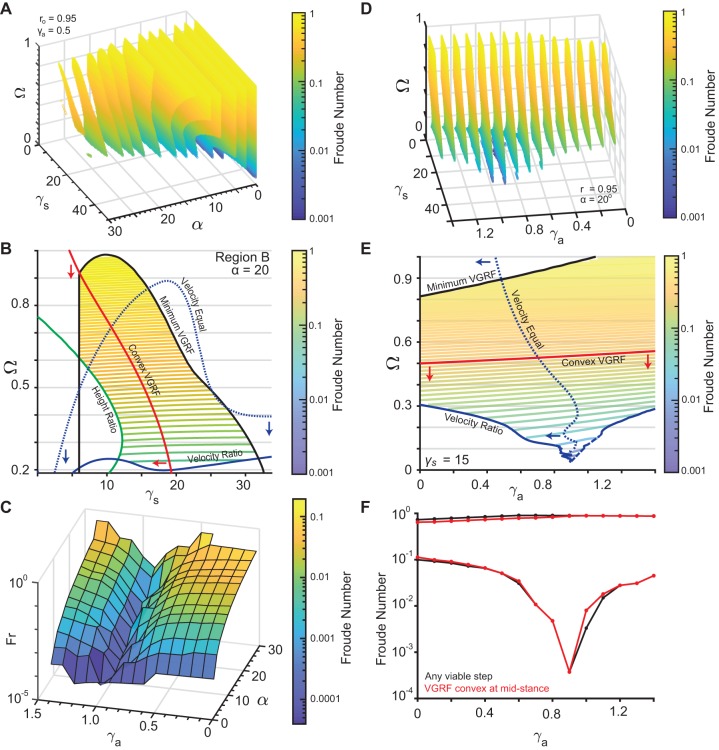
**Non-dimensional analysis of the ARSLIP model.** (A) A sequence of 2D slices each corresponding to one value of *α*. The colored area is allowed under the constraints described in Fig. 1D. Colors represents the Froude number. The vertical mid-stance height *r*˳ is fixed at 0.95 and γ*_a_* set to 0.5. (B) 2D contour plot of the *α* = 20 degrees slice for *r*˳ = 0.95 and γ*_a_* = 0.5 as in A. Similar to Fig. 2B, red is the boundary of the minimum VGRF criterion, blue the velocity criterion and green the height criterion. The dotted line represents the region where the velocity at the end of single-support equals the mid-stance velocity. Red arrows indicate the region where the vertical ground reaction force is minimum, while blue arrows point to the region where the velocity at mid-stance is less than the velocity at the end of single-support. Contour lines represent the Froude number of the allowed gaits. (C) This depicts the minimum Froude numbers for the gaits that meet the *V_r_*, *H_r_* and *G_r_* constraints, plotted against *α* and γ*_a_* with a fixed *r*˳ of 0.95 and minimizing over the other parameters. As γ*_a_* increases, the slowest steps that can be modeled decrease. (D) 2D color-contour plots with color representing the Froude number with Ω, γ*_a_* and γ*_s_* varying, *r*˳ set to 0.95, and *α* set to 20°. Each slice corresponds to different values of the angular spring. (E) 2D slice from D, with γ*_s_* = 15. Same color code as B. The dashed line along the velocity ratio criterion indicates that the boundary is linearly interpolated since the simulation could not resolve the boundary continuously. (F) The range of Froude numbers possible when *α* = 20 and *r*˳ = 0.95 for different values of γ*_a_* with γ*_s_* and Ω free to vary. The black curve represents any steps that meet *V_r_*, *H_r_* and *G_r_*, while the red curve is for the steps that additionally meet the convex VGRF criterion.

The gaitspace for ARSLIP is shown in Fig. 3. Fig. 3A and B show the same plot as Fig. 2A and B, except that now the additional parameter, γ*_a_* is set at 0.5. Fr can reach lower values; for instance, the lowest Fr in Fig. 3B is about 0.02 whereas in Fig. 2B it is about 0.28. As one increases γ*_a_* it becomes possible to achieve progressively slower speeds (Fig. 3C). As noted in Biswas et al. (2018), one way to see this effect is to consider the small angle limit of ARSLIP evolution, |*ѱ*|≪1 and ignore the slight variations in *r*, so that the evolution equation for *ѱ* becomes:(11)
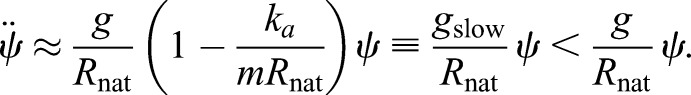
We note that 
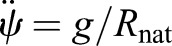
, is the equation for an inverted pendulum rotating under the influence of gravity. Eqn 11 shows that angular springs can counter gravity by effectively making it weaker.

The effect of the angular spring on gaitspace is shown in Fig. 3D. At a given value of *α*, the gaitspace expands as the angular spring becomes stiffer until it reaches a critical value. A similar trend is observed at a fixed value of γ*_s_* (Fig. 3E). This critical value does depend on *α* as can be seen in Fig. 3C; for smaller *α* the critical γ*_a_* is also small. The range of Fr accessible in ARSLIP as a function of *α* is shown in Fig. 3F. This range is much larger than the range in SLIP Fig. 2C. ARSLIP, surprisingly, also improves the upperbound because the tangential forces are able to provide greater control so that the speed – and hence the centrifugal force – does not increase as much as in SLIP when the leg rotates away from the mid-stance. This implies that the VGRF can be slightly larger in ARSLIP as compared to SLIP and the constraint (Eqn 3) is easier to satisfy.

The ability of ARSLIP to produce smooth COM kinematics can equivalently be described in terms of force. In SLIP (or IP for that matter), the leg spring that counteracts gravity also provides a destabilizing horizontal component that speeds up the animal as it moves forward from the mid-stance. In ARSLIP, the angular spring provides an opposing horizontal component that allows an individual to control its increase in speed. Thus, our analysis predicts that the HGRFs in ARSLIP will be smaller than in SLIP and this may address the deficiency that HGRF amplitudes observed in SLIP fits of human GRFs (Lipfert et. al, 2012) are larger than those observed empirically.

Under some parameter conditions, the horizontal component of the angular spring is larger in magnitude than its radial spring counterpart, causing the leg to decelerate from its mid-stance position (‘inverted gait’). The gaitspace to the left of the black curve in Fig. 3B and E corresponds to the regular gait where the speed increases as the animal goes from the mid-stance to the beginning/end, while the region on the right correspond to the inverted gait. Indeed, the inverted gait is observed in some animals such as stick insect and fruit flies (Graham, 1983; Mendes et al., 2013).

### Fitting models to GRFs during walking: SLIP can model the VGRF, but needs ARSLIP to model HGRF

The non-dimensional analysis in the previous section, consistent with findings in Lipfert et al. (2012) and Hubel and Usherwood (2015), show that it is challenging for SLIP to model slow uniform walks. The analysis also shows (see Fig. 3C) that there are two ways to address this issue: in the first, the subjects can reduce the sweep angle, *α*, as they decrease speed, in which case SLIP could continue to be a decent model (Fig. 2A,C). The alternative would be to introduce tangential forces, or γ*_a_*, as in ARSLIP to allow for greater speed control. To distinguish between these possibilities and to assess the general effectiveness of SLIP and ARSLIP models in describing realistic walking dynamics, we fit the single-support phase of human walking to these models.

#### Obtaining experimental data

**Fig. 4.**
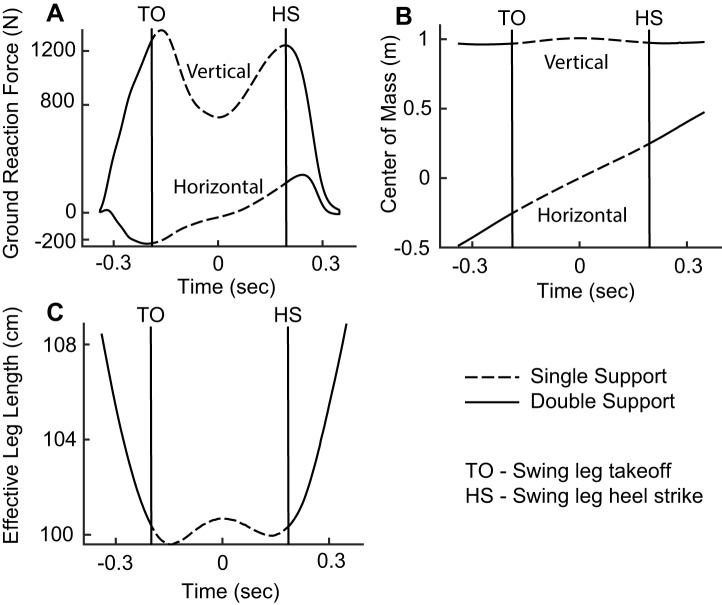
**Single-support data extracted from the experimental trials in preparation for fitting.** (A) Example of the captured ground reaction forces, with the characteristic M-shaped vertical ground reaction force, and the braking and then propulsive horizontal ground reaction force. Dashed lines represent the single-support phase. (B) Example of the captured COM trajectory. The COM rises towards the mid-stance and falls away from mid-stance. (C) The effective leg length as measured from the COM to the projection of the COM at mid-stance to the ground. The compression as the stance leg is loaded during the beginning of the stance phase is followed by a slight decompression towards mid-stance. The leg compresses after mid-stance before unloading in preparation for the swing phase.

Data were acquired as described previously (Hanavan, 1964). Briefly, eight subjects (mass: 74±18 kg, height: 1.69±0.12 m, age: 24±4) walked across four Bertec force plates at a self-selected speed, 20% faster than self-selected speed, 20% slower than self-selected speed and 50% slower than self-selected speed. The force plates were sampled at 1000 Hz and arranged such that the force readings from the left and right leg were each sampled by two force plates each during experimental trials. The coordinate positions of 47 reflective markers on the subject were recorded using the VICON system sampled at 200 Hz. The COM position of each subject during the trial could be calculated from these markers. Single-support phases were extracted from the data (Fig. 4). Runs in which the single-support phase could not be extracted from the data were removed. Effective leg length was calculated as the distance of the COM to the point underneath the COM at mid-stance. The angle of the leg is the angle the COM position makes with the vertical. For each single-support phase, the COM motion was interpolated using a piece-wise cubic Hermite interpolant such that for each GRF sample there is a corresponding COM position.

#### Theoretical fits to experimental data

As noted in the Introduction, our approach differs from others in constraining force, kinematics and stance duration when evaluating a model. In some studies, such as in the virtual pivot models (Maus et al., 2010), only GRFs are considered leading to model parameters that are in clear conflict with the animal's COM motion, as no restriction is placed on the height of the COM. Other studies normalize time-to-stance duration (Buczek et al., 2006), allow stance time to be arbitrary (Lipfert et al., 2012) or only focus on fitting the COM trajectory (vertical height as a function of the horizontal displacement) (Maus et al., 2014).

A Matlab program using the global search algorithm from the Global Optimization Toolbox was written to fit the parameters of the governing differential equations of SLIP and ARSLIP (Eqns 5 and 6) to each single-support phase, minimizing the root mean square error of the predicted GRFs and the sampled GRFs. The leg spring stiffness, *k_s_*, was constrained to lie between 0 and 50,000 N/m while the angular spring stiffness, *k_a_*, could vary from 0 to 5000 Nm/rad. The natural uncompressed leg length, *R*_nat_, was constrained to lie within 10% of the mid-stance leg length. The mid-stance leg length, *R*˳ and angular speed, *ω*, were constrained by the COM data to vary no more than 2.5% and 5%, respectively, from their experimental values. Symmetry around mid-stance (*t* = 0) was assumed in the parameter search such that *ѱ*(0) = 0 and *Ṙ*(0) = 0. Finally, the stance duration was fixed to the empirically observed duration. The best fit parameter was found through gradient descent. Local minimum were avoided by sampling 1000 points within the allowed region. Ignoring points that fell within the same basin of attraction helped to ensure that the search did not get stuck in a local minimum. GRFs were chosen for the objective function over the COM data because of the former's small experimental error, measured in the tenths of a percent. The COM data were used to constrain the possible leg lengths.

The results from these fits are described in the next two subsections.

#### M-shaped VGRF is caused by two cycles of contraction-extension, and to a first approximation, explained by single SLIP

**Fig. 5.**
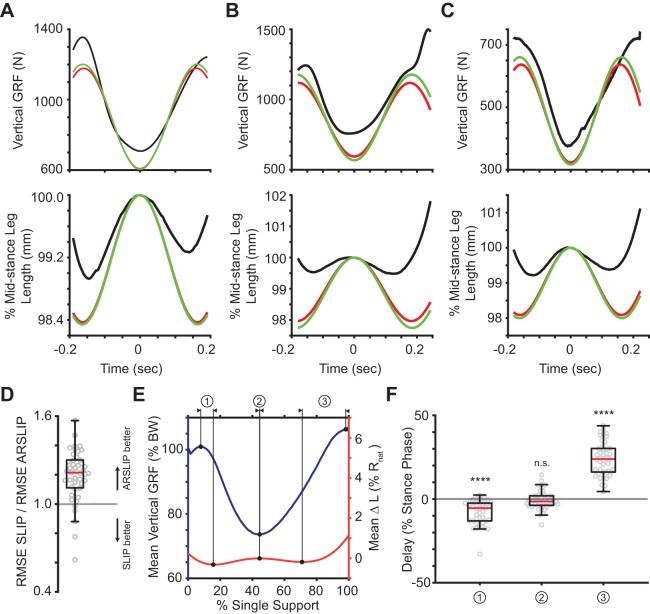
**The M-shaped vertical ground reaction force is due to the leg spring of SLIP.** (A–C) Three example fits from the data showing the relationship between the leg length and the vertical ground reaction force. The change in length of the leg spring drives the M-shaped ground reaction force. (D) While both SLIP and ARSLIP result in similar predictions to the VGRF, but ARSLIP slightly outperforms SLIP in fitting the vertical GRF. (E) The mean vertical GRF and change in leg length for all trials with each trial normalized to its single-support time. Peak vertical GRFs are not aligned well with the minimum leg lengths. (F) The delays between the vertical GRF and the change in leg length for each trial as represented by the previous labels. The first VGRF peak comes before the minimum leg length, while the second VGRF peak comes after the minimum leg length. The minimum VGRF occurs during the local maximum in leg length. ****p<0.0001.

The top panels in Fig. 5A–C shows the empirical VGRF during the single-support phase and the fits of the SLIP and ARSLIP models for three example steps. The bottom panels show the leg length. First, let us take a closer look at the empirical data. Interestingly, the M-shape of the GRF occurs within the single-support phase (Fig. 5A–C, black lines). When averaged across all the steps, the first peak of the VGRF occurs within the single-support phase, and the second peak occurs right at toe-off (Fig. 5E). Importantly, the leg length has a W-shaped profile (Fig. 5A–C, black traces) during the single-support phase. These empirical data are consistent with the idea that the M-shaped VGRF can be modeled through spring-like forces resulting from the W-shaped compression and extension of a single-leg spring. Specifically, the effective leg length undergoes initial compression as the leg is loaded (causing peak VGRFs) and then decompresses (VGRF maximum) as it approaches the mid-stance. After the mid-stance the effective leg length retraces its path leading to an almost symmetric evolution around the mid-stance.

The expected trend above is exactly what we saw when we fit SLIP to the empirical data. The fitted model also goes through a CECE cycle (Fig. 5A–C). ARSLIP provided a small, but significant improvement to the VGRF fit. For the VGRF, the median ARSLIP RMS error is 41.2 N (4.84% of maximum VGRF magnitude), while for SLIP it is 49.9 N (5.95%). Fits to each step in our dataset are shown in Figs S3, S4 and S5.

The fits also reveal a role for active components. If SLIP were a perfect model for the single-support phase, one would expect the peaks and troughs of the VGRF to match the troughs and peaks of the leg length respectively. However, the changes in leg length do not match the timing of changes in VGRF perfectly. This mismatch can be seen in individual examples (Fig. 5B–C), as well as in the average. The plot of average VGRF and leg-length changes demonstrates that the changes in leg length lag the changes in VGRF early in the stance cycle, and lead during the late phase of the stance (Fig. 5E–F).

The large compression of the leg as a person transitions from double-support to single-support [see fig. 2 in Lee and Farley (1998) for example] dominates the length changes of a leg during walking. If one considers the entire stance phase, the leg length is near its minimum during the entire single-support phase. However, if we consider only the single-support phase, the leg length is at its maximum at mid-stance and compresses on either side of this minimum. Both the two minima and the maximum occur during the single-support phase and are partly responsible for the M-shaped GRF. We will show later that this CECE behavior of the leg is important for increasing the distance that the body travels during stance, and for decreasing the velocity redirection during the transition.

#### ARSLIP is a significantly better fit for HGRF than is the SLIP model

**Fig. 6.**
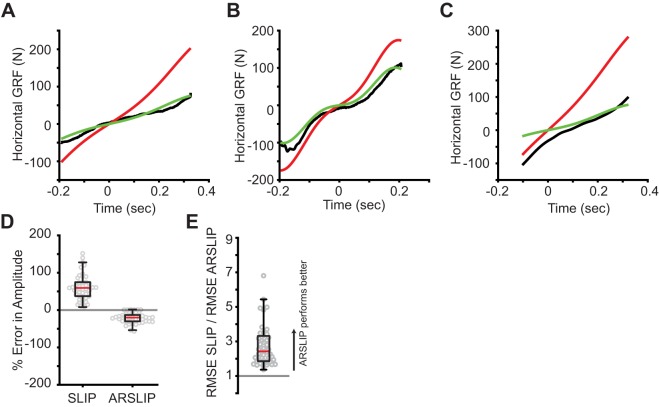
**The angular spring improves the fit in the horizontal direction.** (A–C) Three example fits showing (A) SLIP greatly overestimating the horizontal GRF while ARSLIP makes good predictions, (B) both models capable of fitting more complex HGRFs and (C) SLIP overestimating and ARSLIP underestimating the HGRF. (D) The error in amplitude of the horizontal ground reaction force of the two models. ARSLIP tends to underpredict the amplitude while SLIP tends to overpredict. The magnitude of ARSLIP amplitude error is less than the magntiude of SLIP amplitude error (Mann–Whitney U test, *p*≪0.0001). (E) The ratio of RMSE SLIP to RMSE ARSLIP. ARSLIP performs much better than SLIP in the horizontal direction (sign test, *p*≪0.0001).

Three representative examples of HGRF are shown in Fig. 6A–C. The ARSLIP model (in green) is a much better fit to the empirical data (black) than the SLIP model (red). The best fit of the the SLIP model, as predicted from the non-dimensional analysis, overestimates the magnitude of the HGRF.

During most steps, the HGRF increases linearly. Therefore, the change in HGRF during the step (HGRF amplitude) is a good measure for comparison of empirical data to the models. The HGRF amplitude predicted by the best fit to the SLIP model is larger than observed experimentally (Fig. 6D). The ARSLIP model slightly underestimates the HGRF amplitude, but is a much closer match to the data than SLIP. The median absolute error in the HGRF amplitude for ARSLIP is 38.9 N (20.0%), while it is 114 N (59.4%) for SLIP. The ARSLIP is a much better fit to the data even when the entire time-course is compared. The median root mean square (RMS) error in HGRF for ARSLIP is 21.4 (16.6% of maximum HGRF magnitude), while it is 46.0 N for SLIP (39.8%). Fig. 6E shows the ratios of the RMS errors of the GRFs from the two model predictions. The addition of the angular spring brings the HGRF more in line with experimental data with a median improvement in the RMS by a large factor of 2.4. The probability of SLIP being favored over ARSLIP given the HGRF data fits is less than 0.0001 (sign test).

#### Three mechanisms for speed control: changes in step amplitude, radial spring constant and angular spring constant

**Fig. 7.**
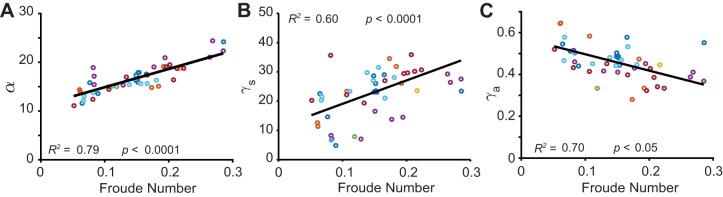
**Speed-dependent change in model parameters.** (A) Maximum angle of sweep decreases with speed. (B) Leg spring constant increases with speed. (C) Angular spring constant decreases with speed. For each linear plot, the *R*^2^ value is for the entire mixed effect model, and the *p*-value corresponds to the significance of the fixed effect slope. Different colors are different subjects.

Non-dimensional analysis suggests that *α* should decrease with speed. Indeed, we observed that while the stance duration increases only slightly with a decrease in speed, the angle of sweep decreases by nearly a factor of two from the slowest to the fastest step (Fig. 7A). The slowest steps, Fr∼0.05, also had the smallest *α*∼11°.

The period of oscillation, *T*, is expected to change as 1/√γ*_s_*, especially for faster motion when the effects of the angular spring is small. Additionally, since ⟨*v*⟩∼1/*T*, this implies that F_r_∼γ*_s_*. Fig. 7B indeed shows evidence of such an overall linear increase (95% confidence interval for fixed effect slope: [54.358, 106.6]). We note that previous studies (Kim and Park, 2011) also recorded increased γ*_s_* with speed.

Based on our gaitspace analysis we expect γ*_a_* to increase as the speed decreases. We observe a similar trend in human data. A mixed linear model showed that the angular spring stiffness increases with a decrease in speed. This is consistent with the notion that the angular spring is needed to prevent large accelerations during slower locomotion (see Fig. 7C).

### Walking in a CECE SLIP cycle contributes to energetically efficient gait

Most mammals walk with an M-shaped GRF and therefore have kinetics that are substantially different from an IP model which produces a concave VGRF with a mid-stance maximum. Simply proposing a compliant leg spring instead of a stiff leg also does not explain the difference because the leg spring employed during walking in SLIP is very stiff, and one would expect its kinetics to approximate that of the IP model. Instead, it generates kinetics that are dramatically different from the IP model because it goes through two contraction-extension cycles (CECE cycle). There are two implications of a CECE cycle.

**Fig. 8.**
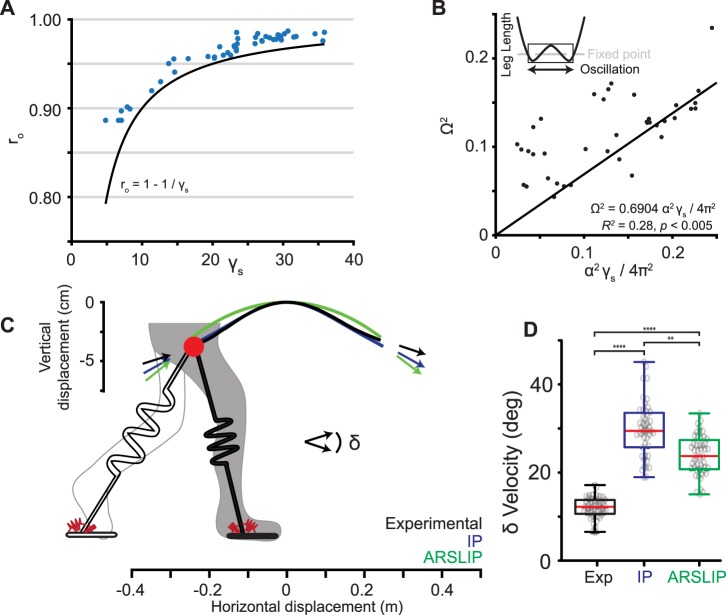
**A springy leg reduces the angle the COM must be redirected to during the double-support phase.** (A) Inverse relationship between the leg stiffness and the non-dimensional leg length, with all experimental mid-stance heights (blue dots) above the predicted fixed point of the spring system (black line). (B) About 70% of the natural oscillation of a spring occurs away from mid-stance during the single-support phase. In other words, the single-support phase is longer than half the natural oscillation period of the leg spring. (C) The trajectory of experimental COM (black), ARSLIP fit to COM data (blue) and inverted pendulum (green). The arrows represent the COM velocity vector at the beginning and end of stance. (D) The distribution of the changes in the velocity angle of the experimental, IP and ARSLIP cases. There are significant differences between the three distributions (Kruskal–Wallis test, *p*≪0.0001). The experimental change in the velocity angle is significantly less than what IP predicts (Dunn-Sidak correction, *p*<0.0001). The spring simulation predicts a smaller change in the velocity direction than IP (Dunn-Sidak correction, *p*<0.005), but greater than what is seen experimentally (Dunn-Sidak correction, ****p<0.0001; **p<0.01).

First, a CECE cycle implies a longer stance duration: At low Ω observed during walking, SLIP functions as a stable oscillator along the radial direction (see Supplementary Information, Approximate time period from dynamics). As a result, the leg oscillates around its fixed point. At the mid-stance the leg length is a little longer than that predicted by the fixed point of the system (Fig. 8A). The expanded mid-stance state implies that the stance phase is lengthened because it needs time to compress before expanding. Empirically observed single-support phase lasts 0.7 of the time period of the springy oscillation (Fig. 8B). The expectation from a CE cycle would be that the stance duration would be 0.5 of the time period (see Supplementary Information, Approximate time period from dynamics). A longer step duration increases the step length α, thereby decreasing the cost of transport because the leg rotates through a longer distance using a conservative spring system.

Second, the CECE walking mode is also more energy efficient in terms of the energy lost during transition. An influential idea in walking is that the single-support phase can be approximated by a passive model such as the inverted pendulum model, and the transition that occurs in the double-support phase is responsible for redirecting the velocity vector (Donelan et al., 2002; Adamczyk and Kuo, 2009). At the end of the stance, COM velocity is directed downwards and has to be redirected upwards at the beginning of the next step. We measured the empirical velocity redirection by assessing the change in velocity vector between the beginning of single-support and the end of single-support. As expected, this change in the velocity vector is small because part of the velocity redirection occurs in the single-support phase itself. We also computed the velocity redirection necessary if the single-support was modeled either by ARSLIP undergoing a CECE cycle or by the IP model. For this purpose, we fit ARSLIP and IP to the individual steps but this time minimizing the COM error (Fig. 8C). Indeed, the velocity redirection necessary was smaller in the ARSLIP model as compared to the IP (Fig. 8D) model. Thus, the mechanics of the single-support phase is better approximated by a CECE of an ARSLIP system than by an IP model (and by extension CE of a SLIP spring).

## DISCUSSION

### SLIP and ARSLIP as a model for walking: insights and limitations

During most steps (43/47), the leg length features a W-shape during the single-support phase, implying that the leg undergoes a CECE cycle. A stiff spring (average stiffness 20 kN/m) undergoing a compression of few centimeters can produce the experimentally observed VGRF. The small length changes underlying the W-shape are dwarfed by the large changes in leg length at the transition between single- and double-support phases. If the stiff spring of the single-support phase were to undergo these dramatic length changes, the resulting VGRF changes would be very large. Thus, the spring constant at the transition must be less stiff, and it is unlikely that the full-stance phase in humans can be described using a single-spring constant. The need for two springs also explains why VGRF fluctuations observed in DSLIP models are usually much larger than empirically observed VGRFs (Hubel and Usherwood, 2015).

DSLIP also produces short stance durations. The W-shaped leg length is one mechanism by which stance duration is lengthened, because the spring undergoes a CECE cycle. The CECE cycle also helps in keeping the COM trajectory relatively flat. Another mechanism that helps lengthen the stance duration is the angular spring of the ARSLIP model. The angular spring increases the stance duration by 5–10%.

Where ARSLIP is absolutely necessary is for producing HGRF forces in which magnitude matches empirical data. However, because some of the transition from the single-support phase to the double-support phase occurs during the single-support phase, passive models are unlikely to capture all the dynamics of even the single-support phase of walking. This limitation is apparent in the small mismatch between the timing of the peaks in the GRF and the peaks in the leg length. In future work, adding a simplified transition mechanism to the passive single-support that is described by a stiff spring going through a CECE cycle would further improve the description here, and would likely serve as a complete model for human walking.

### SLIP works in a mode which supports energy-efficient walking without large changes in COM height

Work that started in the late 1950s in understanding human walking emphasized the importance of walking without large vertical changes in the height of the COM as a mechanism for achieving energy efficiency in walking. Recent work has shown that minimizing vertical movement of the COM does not necessarily result in minimizing work (Gordon et al., 2003; Ortega and Farley, 2005). In fact, one energy efficient model, inverted pendulum (IP), has a diametrically opposite prediction for energy efficient COM kinematics wherein the COM undergoes large changes in height. However, it is clear that the changes in the vertical height of the COM during walking are much smaller than that predicted by the IP model (Lee and Farley, 1998). The general idea that much of the step – except for the short transition period between one step to the next – can be modeled by a passive model is attractive. We show that employing SLIP as the passive model instead of IP can help resolve some of the inconsistencies above and this point is discussed below.

The utility of SLIP as a model for walking partially derives from the fact that it functions as a stable system (see Eqn 32 in the Supplementary Information) that oscillates about the fixed point determined by the system consisting of the leg spring. Importantly, at mid-stance the leg is in its most expanded state and the COM is slightly above this fixed point. From this expanded state, the leg contracts before expanding to its original length. There are three consequences of the expanded leg position at mid-stance on the movement of the animal's COM. First, it increases the duration of the passive phase allowing the animal to travel a longer distance during the passive phase. Second, using a springy mechanism allows much greater control over the speed with which the animal moves through the stance phase. As the animal walks faster, the spring becomes stiffer and the stance duration shorter. Third, the changes in the height of the COM can be smaller than that predicted by the IP model. The change in height of the COM if the leg length is fixed is given by *l*sin(*ѱ*); as *ѱ* increases, the height decreases. The net change in the height of the COM in SLIP is less because at large angles when a fixed-length leg would decrease in height, the leg length is increasing (because of the W-shape of leg length), and partially compensates for the decrease in height due to geometry.

We also show that employing a spring as the passive element will also decrease the amount of velocity redirection that needs to be performed at each step. In sum, the flat trajectory proposed by Saunders et al. (1953), and the passive models proposed later are not as much at odds as it might appear at first glance.

### ARSLIP as a general model for locomotion

This study shows that in the context of human walking, the leg spring is stiff. In comparison, the angular spring is relatively weak. Therefore, the angular spring has a relatively small effect on the kinematics in the context of human walking. The most noticeable contribution of the angular spring is in decreasing the HGRF. The angular spring also makes the stance duration longer, but the stance duration is lengthened by only a few percentage points.

However, the angular spring makes a much larger impact on the locomotion of fruit flies. In the case of fruit flies, the angular spring is much stronger in comparative terms; strong enough that the acceleration due to the angular spring dominates the deceleration due to the leg spring (Chun et al., 2018 preprint) leading to a velocity maximum at mid-stance.

**Fig. 9.**
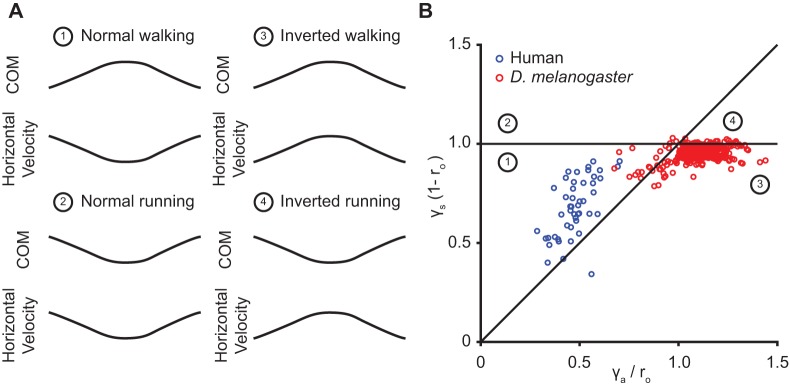
**ARSLIP can produce all four kinematic patterns based on COM and horizontal velocity profile.** (A) There are four possible locomotion types when looking at COM trajectory and the horizontal velocity profile. The walking gait types have a maximum in height at mid-stance, while the running a minimum. The inverted locomotion types have a maximum in horizontal velocity at mid-stance, unlike the minimum that is normally seen. (B) ARSLIP can produce all of these patterns based on the parameters. ARSLIP can capture in one framework the human and fruit fly walking patterns. The numbers correspond to the walking types that were labeled in A.

More generally, based on whether there is a mid-stance maximum in speed or height, there are four kinematic patterns (Fig. 9A). All of these kinematic patterns can be produced by the ARSLIP model. These kinematic patterns represent different regimes of the ARSLIP system. It is possible that ARSLIP represents a general model of legged locomotion. Future studies will test this idea by measuring kinetics and kinematics in many animal species, and a rigorous assessment of how well ARSLIP can model empirical data.

## Supplementary Material

Supplementary information
